# Does Competition Really Bring Out the Worst? Testosterone, Social Distance and Inter-Male Competition Shape Parochial Altruism in Human Males

**DOI:** 10.1371/journal.pone.0098977

**Published:** 2014-07-30

**Authors:** Esther Kristina Diekhof, Susanne Wittmer, Luise Reimers

**Affiliations:** University of Hamburg, Biocenter Grindel and Zoological Museum, Institute for Human Biology, Neuroendocrinology Unit, Hamburg, Germany; University of Vienna, Austria

## Abstract

Parochial altruism, defined as increased ingroup favoritism and heightened outgroup hostility, is a widespread feature of human societies that affects altruistic cooperation and punishment behavior, particularly in intergroup conflicts. Humans tend to protect fellow group members and fight against outsiders, even at substantial costs for themselves. Testosterone modulates responses to competition and social threat, but its exact role in the context of parochial altruism remains controversial. Here, we investigated how testosterone influences altruistic punishment tendencies in the presence of an intergroup competition. Fifty male soccer fans played an ultimatum game (UG), in which they faced anonymous proposers that could either be a fan of the same soccer team (ingroup) or were fans of other teams (outgroups) that differed in the degree of social distance and enmity to the ingroup. The UG was played in two contexts with varying degrees of intergroup rivalry. Our data show that unfair offers were rejected more frequently than fair proposals and the frequency of altruistic punishment increased with increasing social distance to the outgroups. Adding an intergroup competition led to a further escalation of outgroup hostility and reduced punishment of unfair ingroup members. High testosterone levels were associated with a relatively increased ingroup favoritism and also a change towards enhanced outgroup hostility in the intergroup competition. High testosterone concentrations further predicted increased proposer generosity in interactions with the ingroup. Altogether, a significant relation between testosterone and parochial altruism could be demonstrated, but only in the presence of an intergroup competition. In human males, testosterone may promote group coherence in the face of external threat, even against the urge to selfishly maximize personal reward. In that way, our observation refutes the view that testosterone generally promotes antisocial behaviors and aggressive responses, but underlines its rather specific role in the fine-tuning of male social cognition.

## Introduction

The propensity to help and benefit fellow group members, even when incurring costs for oneself (altruism), and an increased hostility towards “outsiders” that are not part of one's own group (parochialism) are behaviors commonly observed in humans [1]. Humans tend to altruistically punish free-riders who commit social norm violations and reward norm-abiding acts even at substantial costs for themselves [2], [3]. They also typically favor genetically unrelated group members (e.g., people from the same linguistic group) over those from a distant outgroup and show increased hostility as well as reduced empathy and trust towards outsiders (e.g., [4]–[7]). As a consequence, norm violations by individuals from a distant outgroup are often more severely punished than those committed in the ingroup, and humans protect group members against outgroup threat even if this incurs costs for themselves (e.g.,[8]).

The emergence of these behaviors in humans is astounding as they are potentially costly or offer no or only minimal benefits for the individual. Evolutionary biologists have recently proposed that this parochial altruism, defined as an increased ingroup favoritism and a heightened outgroup hostility, may represent a crucial step in the evolution of human social behavior [1], [9]. If groups frequently compete over resources or territory, group success often depends on the willingness of individuals to altruistically engage in hostile acts with outsiders, even at risk of high personal costs like death or mutilation [1]. In this context antisocial acts of aggression or non-cooperation that damage outgroup members do protect and support the prosperity of the ingroup and indirectly strengthen internal cooperation. Still, this is different from internal cooperation in the narrow sense, which requires altruistic acts that are directly allocated to members of the ingroup, like altruistically rewarding conformist behavior of fellow group members [2]. Interestingly, the frequency of such altruistic acts that directly stabilize internal cooperation also increases during war [10]. Parochial altruism may thus paradoxically promote both ingroup cooperation and outgroup hostility, which contribute to group success in intergroup conflicts and probably encouraged the proliferation of these expensive group-beneficial behaviors in humans [9].

But what is the proximate driving force of parochial altruism? Albeit cultural influences like community-based sanctions can be one motivation to engage in costly altruistic acts [11], endogenous physiological factors may also predispose individuals for acting altruistically and for discriminating against outsiders. The neurosteroid testosterone has been implicated in the way humans react to competition and intergroup conflict and its various effects include the promotion of social aggression and dominance [12]. Competitive behavior is more amplified in human males, in whom testosterone is one of the dominant steroid hormones, and men are more prone to form coalitions against other men to fight over territory, resources and status [13], [14]. Men with higher testosterone levels also show more acts of retaliatory aggression and are more willing to compete with other men for resources or social status [15]–[19]. Further, male testosterone concentrations tend to rise when social status is threatened [16], [20]. Testosterone also increases the vigilance for hints of social provocation [21], but at the same time diminishes cognitive empathy and interpersonal trust, which is particularly evident during interactions with strangers [22]–[26]. Finally, testosterone acts on the neural circuitry that mediates social aggression (amygdala and orbitofrontal cortex) and augments its responses to stimuli of social threat [27], [28]. Given this evidence, it stands in reason to assume a close relationship between endogenous testosterone and the increased willingness to engage in hostile acts that are targeted at members of the outgroup, particularly during an intergroup conflict. We therefore hypothesize that testosterone may be one proximate candidate for promoting parochial altruism in humans.

However, in assuming that testosterone indeed promotes parochial altruism as a whole, one would also expect a positive relationship between testosterone, ingroup favoritism and behaviors that directly stabilize internal cooperation (e.g., altruistic punishment). Several studies have already examined the potential link between testosterone and altruistic behaviors (see [29] for a recent review). Yet, findings are contradictory. Some studies showed that testosterone may indeed advance altruistic tendencies in social exchange tasks. Accordingly, both endogenous and exogenous testosterone increased the inclination to altruistically punish unfair behavior [30]–[32], and enhanced interpersonal generosity [33], [34]. Nevertheless, other studies reported a significant rise in selfish or antisocial behavioral tendencies that paralleled ascending endogenous and exogenous testosterone levels (i.e., reduced interpersonal generosity [32] and disrupted cooperation in cognitive tasks [35]–[37]), or found no effect of exogenous testosterone on altruistic punishment [33].These conflicting findings may in part be explained by a failure to control for the possibility that testosterone may also be related to parochialism. For example, the observation that the willingness to cooperate with strangers was least pronounced in subjects with high testosterone levels [35] does not come as a surprise considering the potential influence of testosterone on interpersonal trust during interactions with strangers [25]. Further, it cannot be ruled out that differences in the predominant biological sex under research (e.g., male subjects in [32] versus female subjects in [33]) and thus sex differences in androgen effects or aromatase activity throughout the human brain might have resulted in conflicting findings in studies that administered testosterone (see also [38]–[40]). Finally, it is possible that exogenous testosterone may not adequately mimic the physiological effects of endogenous testosterone on cognitive processing (e.g., [41]), or may even reverse or over-exaggerate naturally occurring testosterone effects (e.g., [42]). Even more importantly, exogenous administration only induces a transient change in testosterone levels that may or may not be sufficient to significantly alter behavior. In contrast, endogenous testosterone levels can be considered as a stable hormonal marker of interindividual differences with a high re-test reliability comparable to that observed for some personality variables [43] and thus may be more adequate to demonstrate a hormone-behavior relationship. For these reasons, in the present study we decided to focus exclusively on the behavior-modulating effects of endogenous testosterone in human males.

**Figure 1:**
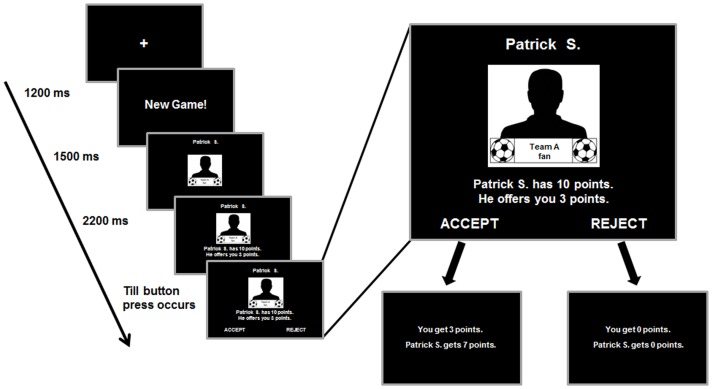
Example of an experimental trial in the computer-based Ultimatum Game (UG). Male soccer fans played the UG as responders and could either accept or reject the offer made by an anonymous proposer. They faced four different types of anonymous proposers: (1.) fans of the respondent‘s favorite soccer team (*ingroup*), (2.) fans of a soccer team rated as neutral (*neutral outgroup*), (3.) fans of an unknown cricket team (*unknown outgroup*), (4.) fans of the soccer team most hated by the respondent (*antagonist outgroup*). Offers made by these agents were either fair (i.e., offering 4 or 5 points out of ten) or unfair (i.e., offering 1, 2 or 3 points out of ten). The UG was played in two consecutive contexts: (1.) without a competition between groups (*neutral context*), and (2.) with an instructed group competition (*competitive context*).

The aim of the present study was to directly assess the potential link between endogenous testosterone and parochial altruism in a group context (soccer) that is characterized by both strong ingroup favoritism and robust rivalry between groups. In particular, we wanted to investigate whether testosterone indeed modulates altruistic punishment tendencies as a means of ingroup norm enforcement or whether this relationship is rather limited to aggression against distant outgroup members when facing an intergroup conflict. Fifty male soccer fans with a strong feeling of group coherence were recruited to perform an Ultimatum Game (UG). The UG is an economic exchange task, in which two players interact [44]. One player acts as the proposer (Player A), who offers a certain share of a fixed amount to the second player (Player B). Proposals can be fair or unfair and Player B has to decide whether to accept or reject an offer. When he accepts the proposal, both players receive their share as offered by Player A. Upon rejection, both receive nothing. The UG is the ideal task to measure costly altruistic punishment in the laboratory. In the UG, humans commonly diverge from economical rationality and do not maximize their total payoff, but tend to punish unfair offers at the expense of their personal reward [44]. This is interpreted as reflecting a norm enforcement tendency necessary to promote cooperation amongst unrelated individuals of the same group [2]. However, the rejection of unfair offers may also reflect an aggressive act in response to social provocation and status threat [31], [45]. Intergroup conflicts often start after a social provocation, which triggers costly retaliatory acts to harm outgroup members. For this reason, one would expect an increase in the rejection rate of unfair offers during interactions between socially distant or rival groups. In our study male soccer fans faced 36 anonymous proposers in single-shot interactions in a computer-based UG (Fig. 1). Proposers were either marked as fans of the same team as the responders (ingroup) or were denoted as fans of one of three other teams (two soccer teams and one cricket team). These latter teams differed in terms of their social distance and enmity to the ingroup (i.e., the neutral, the unknown, and the antagonistic outgroup), which was expected to trigger different degrees of parochialism and outgroup hostility. Proposals made by each group could be either fair or unfair. The UG was played twice in two contexts with different levels of intergroup rivalry. In the neutral environment, subjects played the UG with fans of all other teams and were instructed to maximize their personal outcome. In the competitive context, intergroup rivalry was further escalated to a real intergroup conflict. Responders were additionally told that cooperation with their fellow group members would maximize group reward and would result in extra points if they outperformed the other groups in the competition. However, this also demanded the sacrifice of a fraction of one's personal reward, since group success could only be achieved if subjects also minimized outgroup reward.

**Figure 2:**
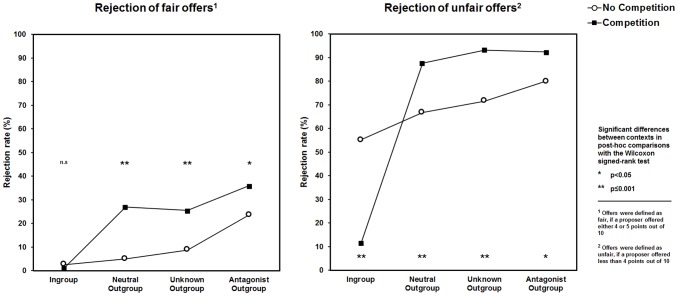
Rejection rates varied with the type of proposal, group membership and experimental context. (A) Rejection rates of fair offers by ingroup and outgroup members. Even in case of fair bargaining rejection rate increased with social distance to outgroup (outgroup hostility) and during the competition context. (B) Rejection rates of unfair offers by in- and outgroup members. Again, outgroup hostility increased with social distance and competition. Except for offers made by ingroup members, in the competitive context the rejection rate of unfair offers decreased drastically.

**Figure 3:**
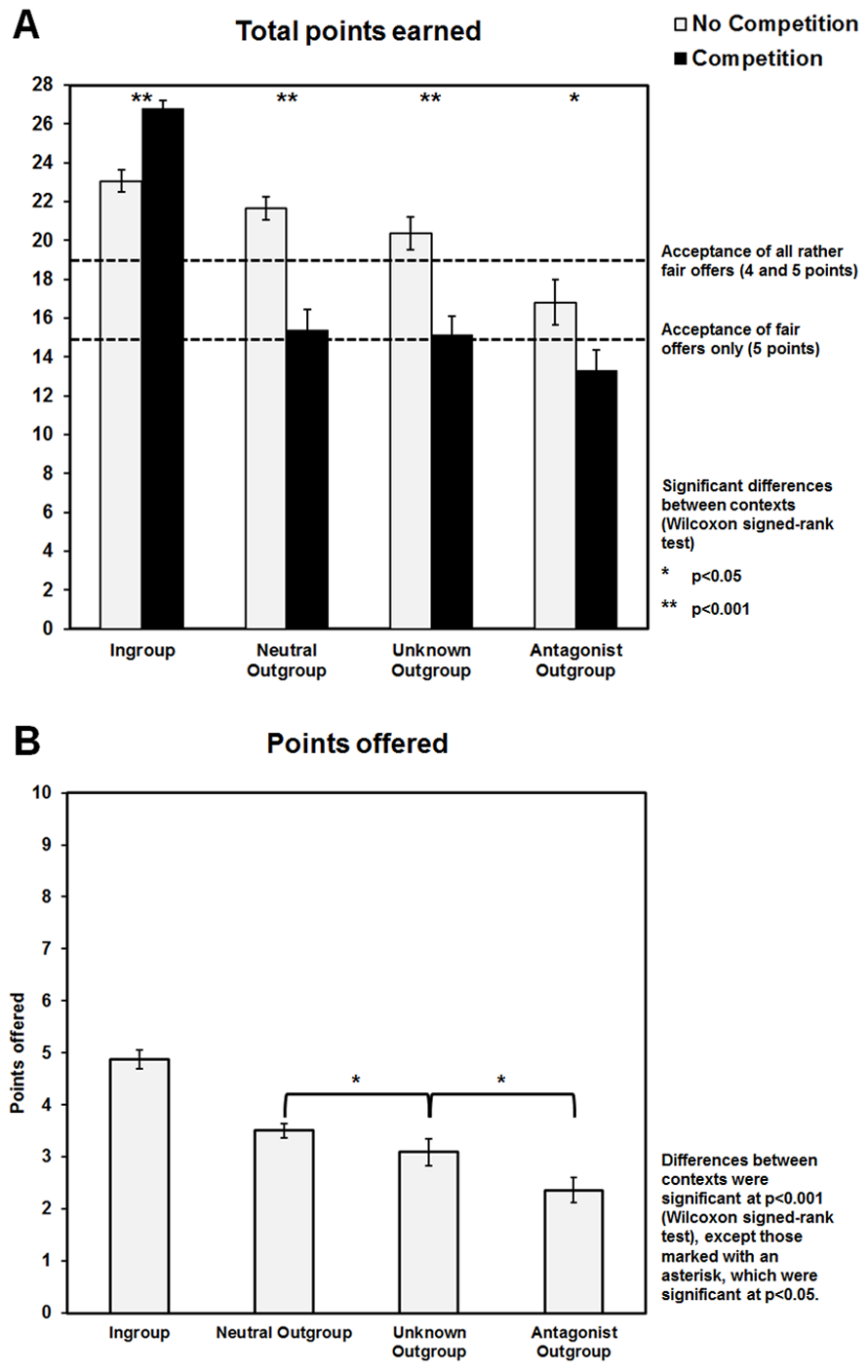
The individual outcome (total points earned) and proposer generosity depended on group membership and context. (A) Subjects earned significantly more points in ingroup interactions and significantly less points in outgroup interactions in the competitive context relative to the neutral context. (B) The number of points offered differed between groups.

**Figure 4:**
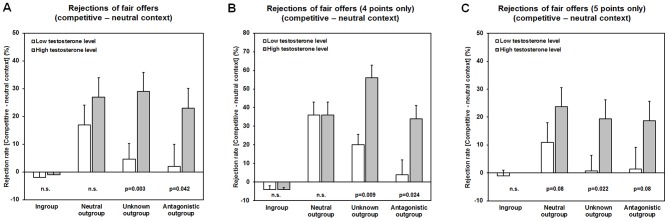
Endogenous testosterone influences modulates behavioral adaptations between contexts. (A) Subjects with high testosterone levels exhibited a significantly stronger increase in the number of rejections of fair proposals, when these were made by a member of the unknown or the antagonistic outgroup (behavioral adaptation between neutral and competitive context). (B) and (C) This was also the case when examining the offer values of 4 and 5 points separately.

The behavioral results indicate that unfair offers were rejected more frequently than fair proposals, and the rejection rates increased with social distance and enmity to the outgroups (ingroup < neutral outgroup < unknown outgroup < antagonist outgroup). In the competitive context, the intergroup bias in the rejection rates was even more pronounced suggesting an escalation of outgroup hostility, while norm-enforcement tendencies in the ingroup were attenuated (i.e., reduced rejection of unfair ingroup offers) (Fig. 2). This was also reflected in the sum of points acquired during the game as total points decreased with increasing social distance indicating a higher rejection rate, especially in the competitive context (Fig.3). Interestingly, endogenous testosterone augmented proposer generosity in interactions with ingroup members in a follow-up email inquiry and also promoted behavioral plasticity towards increased outgroup hostility in the competitive setting (Fig. 4). Collectively, these data support the view of a significant relation between testosterone and parochial altruism in human males. High endogenous testosterone levels predicted increased prosocial tendencies during interactions with the ingroup as well as an escalation of costly outgroup hostility, when subjects competed with other groups.

## Materials and Methods

### Ethics statement

The study was approved by the ethics committee of the medical association of Hamburg (Aerztekammer Hamburg) and subjects gave written informed consent prior to the examination. Participants were paid for participation. They were told that they could win up to 15 Euros depending on the outcome of the UG and of another economic decision task (i.e., a prisoners dilemma), whose results will be reported in another publication. However, in the end all subjects received the total sum of 15 Euros regardless of their outcome.

### Participants

Fifty healthy male soccer fans (mean age ± sem =  24.6±0.5 years; 12 smokers) participated in the present study. Participants were recruited from the student population of the University of Hamburg by advertisements (internet and flyers) and word of mouth. All participants showed an above-average commitment to soccer (Mean score  = 4.06±0.09 on a 5-point-Likert-scale in the rating of the question “*How much are you interested in soccer?*” with answers ranging from 1 “*not much/not at all*” to 5 “*very much (soccer is my life)*”). When being inquired to rate their affiliation/commitment to each of the 18 soccer teams that currently played in the German Premier League (*Bundesliga*) as well as to one local city team that was currently part of the 2^nd^ division, all subjects unequivocally named one favorite and an antagonist team (Question “*How much do you like any of the teams listed below?*”, Rating on a 5-point-Likert-scale from score 1 for “*My favorite team*” to score 5 “*Not at all*”). Participants also rated at least one soccer team from the list as neither bad nor good (i.e., score 3 for “*Neutral*”), which was a further necessary prerequisite for being included in the subsequent behavioral experiment (see below).

### Experimental paradigm

Male soccer fans played two sessions of 36 single-shot interactions as responders in a computer-based UG (see Fig. 1). In a post-test session they then switched to the role of a proposer in four hypothetical interactions. The UG is an economic exchange task, in which two players interact. One player is the proposer (Player A), who offers a certain share (e.g., 3 points) of a fixed sum (e.g., 10 points) to the second player (Player B). Proposals can be fair or unfair and Player B has to decide whether to accept or reject an offer. When he accepts the proposal, both players receive their share as offered by Player A (e.g., Player A gets 7, Player B gets 3 points). But if he rejects the offer, both receive nothing. To fulfill the criterion of economical rationality, Player B would be expected to accept all offers, even the most unfair ones, as this is the only way to maximize the total payoff. Still, humans often diverge from this pattern and tend to altruistically punish unfair offers at the expense of their personal reward. This is interpreted as reflecting a norm enforcement tendency necessary to promote internal collaboration even amongst unrelated individuals [2], [44].

In the present study subjects played the UG as Player B and had to respond to fair and unfair proposals made by anonymous proposers that were fans of different soccer teams. Subjects played 36 interactions with fans of four different soccer teams. Of the anonymous proposers, 9 were marked as fans of the same soccer team as the responder (*ingroup*), 9 proposers favored the team that was most hated by him (*antagonist outgroup*), while the remaining proposals were either made by fans of a soccer team that was rated as neutral (*neutral outgroup*) or by fans of an unknown cricket team (*unknown outgroup*). The respective teams were selected according to the individual rating of the subject's commitment to different soccer teams as described above, meaning that each participant received a personalized version of the task (e.g., Subject 1 might have rated the local city team as his favorite soccer team (*ingroup*), while subject 2 might have classified the same team as *antagonist outgroup*). In the end, data from different subjects thus could be easily combined for each of the four groups.

Of the 9 proposals made, four were fair (i.e., proposers offered 40% or 50% of a fixed total sum (10 points) to Player B), while the remaining proposals were unfair (i.e., proposers offered less than 40%). Accordingly, three proposers offered five and one offered four out of ten points (*fair offers*), while another two proposers offered one point, two offered two points and one offered three points out of ten (*unfair offers*). This classification is in line with previous evidence suggesting that most proposers offer between 40% and 50% of the total sum, which is considered as ‘*fair*’, while offers that lie around and below 30% are frequently rejected because they are regarded as highly unfair [46], [47].

Interactions were presented in a pseudorandomized trial sequence that was counterbalanced for trial-type transitions (i.e., counterbalanced for type of proposal and team membership of the proposers). An individual trial always started with a frame indicating that this was an interaction with a new proposer (“*New Game!*”). Following this introduction in the next frame a silhouette of a young man who faced the responder was shown as a placeholder for the proposer. The first name and last name initial of Player A was superimposed above and the team logo occurred below the silhouette to show the responder that he played with different fans in each trial. In the next frame the offer by Player A was added below the picture, before Player B got the option to respond (i.e., to accept or reject the offer) on the fourth frame. A response made by the responder terminated this frame and the subject received an immediate résumé of his decision before a new interaction with another Player A began (see also Fig. 1 for an exemplary trial and trial timing).

In order to enforce the social nature of the task (see for example [48]) and to make the interactions with the anonymous and actually fictive proposers more realistic, subjects were told in advance that all proposals shown were actually real and had been made previously by another group of soccer fans tested in our laboratory.

The UG was played twice in two contexts, starting with 36 interactions in a neutral environment, followed by 36 trials in a competitive context. In the neutral environment there was no real competition between groups except from the fact that groups already differed in terms of social distance and enmity to the ingroup. In the first session subjects played for themselves and their subjective reward. In the second session (competitive context) responders had the option to collaborate with other members of their ingroup and to maximize ingroup against ougroup reward for extra points in a competition against all other teams. Importantly, in this second session, maximization of ingroup success would have required subjects to reduce selfishness in interactions with outgroup members (i.e., they had to reject unequal offers made by the outgroups in order to maximize group reward) and to control norm-enforcement tendencies in the ingroup (i.e., they had to accept even the most unfair offers for the sake of their group). The overall reward at stake was 5 Euros. Subjects were told that the acquired points were later cashed into real money, including the extra reward that they could receive for winning the intergroup competition. Previous studies have already shown that the UG provokes altruistic punishment independent of whether proposers offered real money, points or even hypothetical rewards [7] or whether they were paid only a percentage of their total earnings [31]. Therefore, the exact conversion factor for individual points was not explicitly explained to the participants, also in order to avoid any counting or calculations of accumulating monetary rewards that could have interfered with decision-making during individual interactions. The exact sum of the extra reward for winning the intergroup competition was not made explicit for the same reasons. However, upon request we told our subjects that it constituted a substantial amount of the overall reward to be won (lying roughly between 15-20%). Interestingly, the participants neither asked for the exact amount of the extra reward to be earned during the competition nor for the actual conversion factor. Further, we also observed that only five of the 50 subjects played completely for themselves in session 1 and one subject shifted to this type of behavior in session 2 (i.e., these players accepted all offers, even the most unfair ones, in the respective sessions). One may speculate that playing an UG with other soccer fans might have reduced the overall importance of monetary outcome due to an increased emotional engagement in this social context, which let feelings of group commitment, rivalry and enmity mainly drive individual decisions.

Directly before starting the first session of the game (neutral context), subjects received a written instruction as follows: “*This is a so called ultimatum game (UG). In the UG two players interact in an economic exchange task. Player A's task is it to share a certain amount of money (in this case 10 points) with Player B. Player B has to decide whether to accept Player A's offer or whether to reject it. Your role in this experiment is the one of Player B. Several previous participants of this experiment play the part of Player A. If you accept Player A's offer, both of you get paid out the shared points proposed by Player A. If you reject the offer, none of you gets any points. Acquired points are later cashed into real money. In all, the experiment consists of 36 single-trial interactions that you play against these previous participants who are anonymous soccer fans.*

*Example: Max has 10 points. He offers you 3 points.*

*Option (a) You accept his offer: Max receives 7 points. You get 3 points.*

*Option (b) You reject his offer: Max receives 0 points. You get 0 points.*

*Comprehension question: Moritz has 10 points. He offers you 4 points. You reject his offer. Moritz receives … points. You get … points.“*

After the first experimental session, in which subjects played for themselves (i.e., the neutral environment), subjects underwent a second test session, in which they faced a competition between groups. Before the competition started, subjects received a second instruction as follows: “*You will now face a second round of the UG. In general, the game is the same as the one played before. (As a reminder: Two players interact in an economic exchange task. Player A's task is it to share a certain amount of money (in this case 10 points) with Player B. Player B has to decide whether to accept Player A's offer or whether to reject it. Your role in this experiment is the one of Player B. Several previous participants of this experiment play the part of Player A. If you accept Player A's offer, both of you get paid out the shared points proposed by Player A. If you reject the offer, none of you gets any points. Acquired points are later cashed into real money. In all, the experiment consists of 36 single-trial interactions that you play against these previous participants who are anonymous soccer fans.)*

*What is new in the second round? In this round of the game you can win extra points if your favorite team gains more points than all other teams.”*

After having read the instructions subjects were encouraged to explain the task in their own words and had the opportunity to ask questions.

Together with the UG participants also completed a version of the Prisoner's Dilemma Task (PD), which was likewise played in the two contexts. The sequence of the two tasks was counterbalanced across participants, but the experiment always started in the neutral environment of the UG and the PD. The results of the PD will be reported in a separate publication. In all, the experiments lasted approximately one hour.

In a follow-up email inquiry, that took place some weeks after the actual experiment was finished, participants were finally asked how many points they would offer if they switched to the role of a hypothetical proposer and encountered fans from each of the 4 teams. Subjects received the following email text: “*For our experiment we need additional proposals from previous participants. We would very much appreciate, if you answered the following questions: Imagine that you get 10 Euros. How many Euros would you share with Player B? Remember also, that Player B has the opportunity to reject your proposal (if he feels it is too unfair). Then the two of you will get nothing. These are the four teams to whose fans you can make an offer. Please indicate how much you would like to propose: …*”. In total, 41 subjects answered to this email.

### Saliva collection and analysis

At the day of testing, subjects provided samples of their morning saliva to determine a proxy of the free, bioactive testosterone concentration. Starting at normal wake-up time participants collected five samples every 30 minutes over 2 hours in 2 ml Eppendorf tubes. During this time they were not allowed to eat, smoke or drink beverages like coffee, milk or juice to avoid any contamination or dilution of the samples. Tab water to rinse the mouth or to drink was allowed between collection intervals until 5 minutes before each sample collection. Subjects could also brush their teeth directly after the first sample was collected, but had to wait at least 15 min. before the next collection interval started. This was done to avoid potential contamination by blood from brushing-induced micro-lesions. Importantly, this collection of several samples over time controlled for the episodic secretion pattern of steroid hormones [49] and thus ensured that all participants provided a representative sample of their current free testosterone level.

Saliva samples were frozen at −20°C until all participants had completed the experiment. They were then thawed and separated from mucins and other residuals by centrifugation at RCF 604×g for 5 minutes (i.e., 3000 rpm in a common Eppendorf Minispin centrifuge).The five samples were then combined to an aliquot by extracting an equal volume of the clear colorless supernatant from each of the five Eppendorf tubes (at least a volume of 100 µl was extracted from each tube depending on the filling level of tubes). Any samples containing traces of blood were discarded. Saliva samples were then analyzed for testosterone concentrations with an enzyme-linked immunosorbent assay kit (ELISA) purchased from Demeditec Diagnostics (Kiel, Germany) in our in-house laboratory. The lowest analytical detectable level of testosterone that can be distinguished from the zero standard with this assay is 2.2 pg/ml at the 2 SD confidence limit. Samples were assayed twice (mean coefficient of variation of all measurements  = 5.75%, sem  = 0.87%, n = 30). Further, two control samples (high and low control samples) were also run in the assay. Since the sample size precluded an analysis of all samples with one ELISA, two assays were used on two different days (assay 1: n = 30; assay 2: n = 20).

### Behavioral data analysis

Behavioral data were analyzed with SPSS/PASW 18. A repeated-measures general linear model (GLM) was performed including the factors ‘Team’ with four levels (ingroup, neutral outgroup, unknown outgroup, antagonist group), ‘Proposal’ with two levels (fair and unfair), and ‘Context’ with two levels (neutral and competitive). Post-hoc comparisons between conditions were performed with the Wilcoxon signed-rank test. P-values smaller than 0.05 were considered as significant (two-tailed significance). Finally, the median of salivary testosterone levels was used to subdivide subjects into two groups of individuals with either higher or lower testosterone levels that significantly differed between groups. This median split was performed separately for assay 1 and 2. Afterwards data were combined and the group means were determined (mean ± sem of high level T = 149.88±6.28 pg/ml; n = 25; mean ± sem of low level T = 94.04±3.63 pg/ml; n = 25). The two testosterone groups significantly differed in their mean testosterone concentration (z = 5.79, p<0.001) (please see [30] for a similar procedure). Comparisons between the two testosterone groups were performed using independent-samples U-tests. Behavioral data are made available as Supporting Information (Table S1).

## Results

### Rejection rates for unfair and fair offers vary as a function of group affiliation and intergroup conflict

We first examined how group affiliation, type of offer and intergroup bias in a competition may affect the overall rejection rate. Unfair offers were rejected more frequently than fair proposals (‘Proposal’: F_(1,49)_ = 298.24, p<0.001), and rejection rate increased with increasing distance and enmity to the outgroup (‘Team’: F_(3,147)_ = 111.01, p<0.001), and there was also a significant main effect of context (‘Context’: F_(1,49)_ = 5.50, p = 0.023) (see also Fig. 2 for the results from the direct comparisons). A comparison of the two contexts further revealed a significant difference in the rejection rates with regard to type of proposal as a function of group affiliation (‘Team × Proposal’ F_(3,147)_ = 38.08, p<0.001), and the different groups in the two contexts (‘Team × Context’: F_(3,147)_ = 52.18, p<0.001). The interaction of the two types of proposals in the two contexts only reached statistical trend level (‘Proposal × Context’: F_(1,49)_ = 3.40, p = 0.071). Most notably we also found a significant three-way interaction of ‘Team × Proposal × Context’ (F_(3,147)_ = 28.32, p<0.001). This interaction was mainly accounted for by a marked rise in the rejections of rather fair offers made by the three outgroups and a significant drop of the refusals to accept unfair offers when interacting with members of the ingroup in the competitive context (Fig. 2; see also Table 1 for additional information on direct comparisons for the individual points offered). Referring to the issue of 4 point offers possibly being considered as rather unfair by some subjects, we ran another GLM that used a more strict classification of proposals into fair and unfair offers: 50% shares (i.e., 5 points out of 10) were classified as fair, whereas <50% shares (i.e., 4, 3, 2, and 1 point out of 10) were considered as unfair. The GLM revealed the same results considering main effects and interactions. We still observed significant effects of ‘Context’ (F_(1,49)_ = 6.24, p = 0.016), ‘Proposal’ (F_(1,49)_ = 266.34, p<0.001) as well as ‘Team’ (F_(3,147)_ = 85.93, p<0.001), which were of the same strength and pointing into the same direction as in our previous analyses. Moreover, the interactions between ‘Team × Context’ (F_(3,147)_ = 42.68, p<0.001), ‘Team × Proposal’ (F_(3,147)_ = 51.71, p<0.001) as well as the three-way interaction between ‘Team × Context × Proposal’ (F_(3,147)_ = 33.77, p<0.001) remained highly significant. Solely, the interaction between ‘Proposal × Context’ disappeared. Yet, according to our initial proposal classification this association has also been found to only reach trend level. Collectively, these observations may reflect an escalation of outgroup hostility in the competitive context as well as increased internal collaboration with reduced norm enforcement tendencies in the same context, which was necessary to maximize overall ingroup reward.

**Table 1:** Percentage of rejections in the neutral and in the competitive context.

Type of offer	Neutral context (Rejection rate in %, mean ± sem)	Competitive context (Rejection rate in %, mean ± sem)	Wilcoxon test
**FAIR – INGROUP: Rejections of fair proposals made by the ingroup**			
5 points	0.5±0.5%	0%	n.s.
4 points	8.0±3.8%	4.0±2.8%	n.s.
**UNFAIR – INGROUP: Rejections of unfair proposals made by the ingroup**			
3 points	34.0±6.8%	12.0±4.6%	p = 0.005
2 points	52.0±6.8%	11.0±4.4%	p<0.001
1 point	69.0±6.2%	12.0±4.2%	p<0.001
**FAIR – OUTGROUP: Rejections of fair proposals made by the three outgroups**			
5 points	neutral: 0.5±0.5%	neutral: 17.8±5.2%	p = 0.003
	unknown: 5.5±2.9%	unknown: 15.5±4.6%	p = 0.041
	antagonist:16.0±4.9%	antagonist: 26.0±5.7%	n.s.
4 points	neutral: 18.0±5.5%	neutral: 54.0±7.1%	p<0.001
	unknown: 16.0±5.2%	unknown: 54.0±7.1	p<0.001
	antagonist: 46.0±7.0%	antagonist: 65.±6.6%	p = 0.021
**UNFAIR – OUTGROUP: Rejections of unfair proposals made by the three outgroups**			
3 points	neutral: 44.0±7.1%	neutral: 82.0±5.5%	p<0.001
	unknown: 50.0±7.1%	unknown: 90.0±4.3%	p<0.001
	antagonist: 64.0±6.9%	antagonist: 84.0±5.2%	p = 0.008
2 points	neutral: 66.0±5.6%	neutral: 88.0±3.9%	p = 0.001
	unknown: 71.0±5.7%	unknown: 93.0±3.5%	p = 0.0.003
	antagonist: 79.0±5.5%	antagonist: 94.0±3.1%	p = 0.017
1 point	neutral: 79.0±5.5%	neutral: 90.0±4.0%	n.s.
	unknown: 83.0±5.3%	unknown: 95.0±2.9%	n.s.
	antagonist: 87.0±4.7%	antagonist: 95.0±2.6%	n.s.

### The total outcome of proposer-responder interactions in the UG (total points acquired) and proposer-generosity are affected by the intergroup bias

We were also interested in the overall outcome of all single-shot interactions in each context (i.e., the total points earned). Consistent with the assumption that intergroup conflict increases internal collaboration in the competitive context subjects exhibited an increased willingness to sacrifice part of their personal reward for the ‘greater good’ of the ingroup, which was reflected by a significant decrease in the number of total points acquired (Main effect of ‘Context’: F_(1,49)_ = 11.25, p = 0.002; neutral context (mean ±sem)  = 81.88±2.55 points; competitive context (mean ±sem)  = 70.54±3.00 points; Post-hoc comparison: z = 3.10, p = 0.002). This competition-related decline was largely driven by the switch toward increased outgroup hostility in the competitive context. The significant ‘Team × Context” interaction (F_(3,147)_ = 32.01, p<0.001) was explained by a marked decrease of points acquired for individual interactions when interacting with members of the three outgroups in the competitive setting (see Fig. 3A). We also observed a significant increase of points earned in ingroup interactions, supposedly reflecting increased ingroup collaboration and tolerance for unfair proposals (Fig. 3A). However, the increased acceptance of ingroup unfairness was insufficient to compensate for the personal loss resulting from interactions with members of the three outgroups, meaning that it probably did not reflect a selfish motivation, which could have been assumed otherwise. Even independent of experimental context we observed a significant decline of the total points with increasing distance and enmity to the team (Main effect of ‘Team’: F_(3,147)_ = 69.44, p<0.001), whereby most points were earned in interactions with ingroup members and the least points were gained when interacting with proposers of the antagonistic outgroup (Fig. 3A).

Finally, the behavioral tendency to treat fellow group members better than those from the three outgroups was not only restricted to the role of being a responder. Having switched to the role of a proposer after completing the UG, our subjects also exhibited an increased generosity towards members of the ingroup (Main effect of ‘Team’: F_(3,120)_ = 32.83, p<0.001) (Fig. 3B).

### Endogenous testosterone affects ingroup collaboration and outgroup hostility in the competitive context

In the final and most important step of the analysis we examined the association between salivary testosterone concentration and the observed intergroup bias. Since we predicted that endogenous testosterone may increase the intergroup bias and may promote outgroup hostility as well as internal cooperation, one-tailed tests were used to test the hypotheses.

In line with our prediction that individual testosterone levels paralleled the degree of the intergroup bias we observed a significant relationship between testosterone and behavioral adaptations between the two contexts. Accordingly, a significant increase of the rejection rate of fair offers (4 or 5 points) that were made by the two most distant outgroups in the competitive relative to the neutral context in participants with high testosterone levels could be found (Fig. 4). This relative enhancement of the intergroup bias during the competition became evident when subjects dealt with the unknown outgroup (Delta of rejections of fair offers: high level T = 29.0±6.9%; low level T = 4.7±5.6%; z = −2.82, p = 0.003), and also when they interacted with fans of the antagonistic soccer team (Delta of rejections of fair offers: high level T = 23.0±7.1%; low level T = 2.0±7.9%; z = −1.73, p = 0.042). In addition to that, we also observed that during the competition the difference in the rejection rate between rather fair offers made by the antagonistic outgroup and the ingroup (i.e., the *Delta of rejections of fair offers (antagonistic outgroup –ingroup)*) was significantly higher in subjects with high T (43.0±7.6%) as compared to those with low T (27.0±6.9%) (z = 1.72, p = 0.043). This became particularly evident in the difference in the offer of 4 points (high level T = 72.0±9.2%; low level T = 50.0±9.6%; z = 1.72, p = 0.043). This suggest that endogenous testosterone levels may have also influenced how subjects differentiated between the ingroup and the most distant outgroup (i.e., increased ingroup favoritism and outgroup hostility) when being confronted with these rather fair outgroup offers in the competitive context. Finally, high testosterone levels were also associated with an increased ingroup generosity. This became evident when subjects switched to the role of a hypothetical proposer. Accordingly, subjects with high testosterone levels offered significantly more points to fans of their favorite team than those with low testosterone levels (Points offered to ingroup team members (mean ± sem): high level T = 5.14±0.22 points; low level T = 4.58±0.26 points; z = 1.77, p = 0.038).).

## Discussion

The aim of the present study was to assess whether endogenous testosterone shapes parochial altruism in human males, and, more specifically, to examine how testosterone influences altruistic behavior during an intergroup conflict. Two major findings emerged: Firstly, subjects with high testosterone levels exhibited a change towards increased outgroup hostility during the intergroup competition, whereas those with low testosterone concentrations acted rather selfishly in the competitive setting (Fig. 4). This suggests that, when group reward was at stake, endogenous testosterone apparently reduced individual selfishness and decreased the inhibition to punish outsiders at one's own expense. Further, in the competitive context subjects with higher testosterone levels differentiated more between the ingroup and the most distant outgroup when facing rather fair offers and were also more generous after the competition when interacting with a member of the ingroup. Taken together these findings fit well with the idea that endogenous testosterone may be positively related to both enhanced ingroup favoritism and outgroup hostility. Secondly, we also observed that parochial altruism already emerged in a situation in which the social distance and enmity between soccer fans was the only group differentiating factor. Rejection rates related to both unfair and fair offers increased with increasing social distance, while the number of acquired points decreased (Figs. 2 & 3A). This means that men showed a general propensity for parochialism that manifested itself in the degree of altruistic punishment tendencies in the UG. But in the absence of an intergroup conflict, namely in the neutral environment, an association with endogenous testosterone could not be demonstrated. Given this evidence, it follows that only the escalation of intergroup conflict by the competitive setting and the explicit requirement to maximize group reward revealed the interdependence of parochial altruism and endogenous testosterone.

What is the implication of these findings? For one thing, the present data are consistent with the notion that testosterone may promote the coexistence of both ingroup-oriented prosociality and antisocial behaviors targeted at outsiders (i.e., male parochial altruism), but only in the context of a group competition. It has been proposed that parochial altruism may intensify in times of frequent conflict [1], [9] and our data demonstrate that this intensification may to some extent depend on endogenous testosterone levels. In the competitive context of our economic exchange task participants with high testosterone levels showed a noticeable behavioral change toward enhanced outgroup hostility (Fig. 4) and were also more selective when responding to rather fair offers of the antagonistic outgroup relative to their ingroup. What is noteworthy about these findings is that high testosterone subjects were actually willing to altruistically sacrifice part of their personal gain for augmenting the probability of winning an extra group reward. This observation clearly refutes the view that testosterone may generally promote antisocial behaviors or aggressive responses [37], but underlines the rather specific role of this hormone in the fine-tuning of male social cognition. Previous studies that used economic exchange tasks revealed a similar coincidence of ingroup favoritism and costly outgroup hostility during intergroup competitions. Bernhard et al. (2006) [8] observed that unfair offers in a dictator game with a third-person punishment option were most harshly punished by an observer when the unfair outgroup member interacted with a member of his ingroup. Also, Campanha (2011) [7] found that friendship significantly modulated refusal rates in the UG, resulting in fewer rejections of unfair offers during interactions with a friend compared to those with a stranger. Finally, in a prisoner's dilemma with the option of third-person punishment the introduction of an intergroup competition, similar to the one employed here, led to enhanced ingroup cooperation and also increased outgroup hostility (i.e., altruistic punishment) [50]. Our study is the first to show that endogenous testosterone may be implicated in the various aspects of male parochial altruism and may determine the extent of adaptive behavioral responses in a competitive setting. By enhancing the willingness to engage in expensive antisocial acts that damage outsiders (i.e., punishment of rather fair outgroup members), endogenous testosterone may have thus indirectly contributed to overall group success and supported the prosperity of the ingroup. This is in agreement with a recently proposed evolutionary theory on male typical intergroup adaptations (i.e., the ‘male warrior hypothesis’; [14]), according to which men, in whom testosterone is the dominating hormone, should be more willing to enter a competition with other men and should be more cooperative under conditions of intergroup threat ([51], see also [13] for a comprehensive overview).

Apart from that, we also found evidence for an involvement of endogenous testosterone in the promotion of behaviors that directly promote internal cooperation. In the follow-up email inquiry, in which we asked our participants how many points they would offer in encounters with members of the four different groups, high endogenous testosterone levels at the day of testing predicted an increased adherence to ingroup fairness norms, which conforms with the observation that endogenous testosterone may represent a stable hormonal marker comparable to a personality trait (see [43]). Men with high testosterone levels indicated the readiness to share a significantly higher amount of points with fellow-group members than men with low testosterone concentrations (see also [30] for a similar observation). It has been suggested that the adherence to ingroup fairness norms acts as a means to promote ingroup cohesion and to ensure future reciprocity, which strongly depends on one's own reputation as a good cooperator [2], [3], [52]. Decisions in human societies and groups follow the principle of generalized exchange (i.e., there is no direct reciprocation of benefits between interacting partners, but people who provide favors can expect to receive benefits from others of their group in return), and people in a group context tend to behave accordingly, because this is the only way to become and stay part of the ingroup (see [53]). From this perspective, our results would imply that the increased ingroup generosity of high testosterone men could have been driven by an automatic behavioral mechanism that increases an individual's reputation and thus the probability of future reciprocity, which would support ingroup cohesion in comparable real-world situations. Alternatively, making a rather fair offer could have also reflected a selfish response, because rejection of relatively fair offers is more unlikely. However, our subjects in general distinguished between the in- and outgroup in that the amount offered decreased with increasing social distance (Fig. 3B). Since participants with high testosterone concentrations also showed an increased willingness to engage in costly behaviors to damage outsiders in the competitive setting, we would therefore rather opt for the first alternative when interpreting the present observation.

Moreover, testosterone has previously been found to increase the sensitivity for signals of social provocation and may mediate responses to social threat [15], [16], [19], [21]. Altruistic punishment may represent a functional response to a status threat [45]. It has been assumed that men, who rigorously punish norm-violators, signal personal toughness (heroism) and social dominance. This is thought to enhance the punisher's social status and reputation within a group, and may thus ensure the retention of alliances during hostile interactions with other groups ([11], [31], see also [10]). Given this evidence, it follows that in the present study endogenous testosterone should have been expected to increase the responsiveness to social provocation in male-male interactions in general, and consequently should have led to more rejections of unfair offers. However, we found only limited evidence for this assumption. Participants with high testosterone levels showed an increased readiness to reject rather fair, but nevertheless unequal offers (i.e., 4 points), but only if these were made by outgroup members and occurred in the competitive context of our task (Fig. 4). Otherwise, we found no indication for a general testosterone-mediated shift in the perception of inequality. This suggests that testosterone may have specifically affected the sensitivity to proposals made by outgroup members, but only in a setting in which subjects tried to maximize overall group reward.

In a wider sense, the present data also do not provide evidence for an interdependence of endogenous testosterone and altruistic punishment per se. In the neutral context, the mere interaction with unfair strangers from either the ingroup or a socially distant outgroup was not sufficient to demonstrate the interdependence of habitual testosterone levels and altruistic punishment. This was the case despite evidence for a significant influence of parochialism on rejection rates in the UG (i.e., increased rejection of outgroup offers) and a general tendency for increased altruistic punishment of unfairness (Fig. 2). Two previous studies found a weak positive relationship between endogenous testosterone and altruistic punishment in the UG [30], [31]. Yet, these studies differ from the present one in some important aspects. Firstly, in the study by Burnham (2007) [30] male responders faced two offers, one highly unfair and one overly fair offer (i.e., $5 versus $25 out of $40). Six subjects rejected the unfair offer, while the majority of 20 participants accepted it. ‘Rejecters’ thereby had an average testosterone level that was by 50% higher than that of the ‘accepters’, a difference that is comparable to the one found in our testosterone groups. Nevertheless, the small number of participants who rejected the unfair offer makes it possible that population outliers may have driven the observed behavioral effects. Secondly, Mehta & Beer (2009) [31] reported a linear increase of rejection rates that paralleled increasing testosterone levels in both men and women (β = 0.35, p<0.05). In their study, monetary offers were made by anonymous proposers whose gender was not indicated. The authors found a much lower average rejection rate (54.06%) for unfair offers than observed in our study (mean rejection rates of unfair offers in the neutral context: overall rejection rate  = 68.4%; ingroup  = 55.2%; neutral outgroup  = 66.7%; unknown outgroup  = 71.6%; antagonist outgroup  = 80.0%). We cannot rule out that either the use of a weaker stimulus (points rather than money) or the fact that in our study interaction partners were clearly delineated as being male and from different social groups may have significantly increased the general willingness to reject unfair offers independent of testosterone levels. This could have led to a behavioral ‘*ceiling effect*’, which might have concealed the already weak relationship between endogenous testosterone and altruistic punishment in the neutral setting.

It is further important to note that group success in an intergroup conflict may depend on several factors. One is the enhancement of internal cooperation by increased altruistic punishment. There is already evidence that groups with internal altruistic punishment commonly exhibit higher levels of internal cooperation and tend to outperform groups without this option, especially when facing an intergroup conflict ([11], [54] see also [2]). This led to the hypothesis that normative behaviors (e.g., fairness in the UG) should be more rigorously enforced in times of war, which has also been supported empirically [10]. At first glance, our observation of a significant reduction of altruistic punishment of ingroup members, that occurred in the context of a group competition (Fig. 4), clashes with this view. Yet, enhanced norm-enforcement tendencies and altruistic punishment in the ingroup may only be one means to consolidate internal cohesion. In our study, an increased tolerance towards unfair ingroup offers was a necessary prerequisite to win the challenge in the competitive setting. Reduced rejection rates in ingroup interactions therefore may have reflected a functional response within the restricted boundaries of our task. Participants also clearly differentiated between groups, in that unfair outgroup proposals yielded significantly higher rejection rates on average when comparing the competition and the neutral setting (rejection of unfair outgroup proposals (mean ± sem): neutral context  = 72.8%±4.5%; competition  = 91.1%±2.9%; z = −3.76; p = 0.001) (see also Fig. 2), which meant that subjects indeed sacrificed personal points to win the group competition. Taken together, these findings clearly refute the assumption that a reduced willingness to reject unfair ingroup proposals during the competition may have reflected a rather selfish strategy of personal reward maximization. Instead, they might again underscore the specific role of endogenous testosterone in the promotion of behaviors that support the prosperity of the group during a conflict.

As a last point, it was very interesting to note that participants evidently differentiated between groups in a way that depended on social closeness and enmity to their favorite team and already did so in the neutral setting (i.e., rejection rate for unfair offers: ingroup < neutral outgroup < unknown outgroup < antagonist outgroup) (see Fig. 2). The same order of increasing rejection rate with increasing social distance was found after applying a more strict classification of proposals into fair (i.e., only offers of equal share) and unfair (i.e., offers <50%). Hence, the parochial nature of altruistic punishment could still be observed after controlling for subjects that possibly considered 4 out of 10 points offers as rather unfair proposals. Other researchers have observed similar group-discriminatory decisions in economic exchange tasks [7], [50]. These observations clearly violate the assumption of economic rationality, according to which no such differentiation between groups should occur when the major goal is to maximize personal outcome. Factors like social distance and hence increased outgroup discrimination most likely explain the emergence of economic irrationality in our study. Outgroup discrimination is a common feature of segmented societies, in which people are divided in different groups that are characterized by strong internal coherence, but increased enmity between each other [55]. The context of soccer fandom and fandom in other team sports possesses many of the qualities that define segmented societies. Sports teams form brands and sports fans represent brand communities with clear-cut borders between them. Fans commonly show a strong emotional commitment to their sports team of choice (see also [56]). Reservations and resentments against followers of other teams increase with rising interest, pride, and loyalty to the favorite team (see also [57]) as do trust and positive feelings for other fans of this team [58], [59]. In its most extreme form, fan membership may even determine the degree of empathy one has for another person. In one study, soccer fans exhibited less empathetic responses and were less willing to help a fan of a rival team, who experienced pain, compared to watching a fan of their favorite team in pain [60]. This observation strongly resembles other findings made in an ethnic context [6], [61]. Being a committed sports fan may thus be comparable to holding a ‘tribal social identity’ (see also [62]), with high levels of ingroup identification and strong hostility toward rival fans. By including only subjects that clearly indicated a favorite and an antagonistic soccer team and who reported a considerably high interest in soccer (i.e., high rating scores for the statement “*soccer is my life*”), we ensured a strong team affiliation and a high degree of sympathy for the preferred team, as well as a significant aversion against the most hated team. In contrast to minimal group paradigms (e.g., [63]), in which arbitrary classifications determine group membership (e.g., perceptual discrimination accuracy) and emotional engagement is minimal, our approach allowed us to study the parochial nature of altruistic norm enforcement in a situation similar to real-world conflicts often faced by rival groups (see also [8]). Against this background it also seems plausible why soccer fans quite strongly and irrationally discriminated against outgroup members, even at the expense of their personal outcome and already in a neutral setting without a group competition. Unlike other studies (e.g., [7]), subjects did not personally know the proposers they interacted with. Still, their behavior suggested increased social closeness to some proposers as unfair ingroup offers were less often rejected than unfair outgroup offers. It thus appears as if it was sufficient for the responder to know that an anonymous proposer was a fan of his favorite team to treat him like a potential ally in conflict.

Taken together, we were able to demonstrate a significant relation between endogenous testosterone level and parochial altruism in human males, but only in the presence of an intergroup competition. Our data thereby provide initial evidence that testosterone may be one endogenous physiological factor to promote internal collaboration and to increase outgroup hostility in the face of external threat, even against the urge to selfishly maximize personal reward. Future studies have to further examine the causality of this relationship with the present experimental design, for example by comparative studies using men with hormonal disorders (e.g., with hypogonadism or Kallmann syndrome) or by administering testosterone to men in a double-blind placebo-controlled fashion like in [32]. As a closing remark, we would like to complete with a quote by the famous US tennis player Arthur Ashe, who stated that *“True heroism is remarkably sober, very undramatic. It is not the urge to surpass all at whatever cost, but the urge to serve others at whatever cost.”*, and endogenous testosterone appears to be one important driving source of this type of prosociality in human males.

## Supporting Information

Table S1 **Behavioral Data.**
(PDF)
