# Supplementary material for: Does Competition Really Bring Out the Worst? Testosterone, Social Distance and Inter-Male Competition Shape Parochial Altruism in Human Males
Source: PLoS One. 2014 Jul 30;9(7):e98977. doi: 10.1371/journal.pone.0098977 (PMC4116333; doi:10.1371/journal.pone.0098977)
Supplement: Table S1 — Behavioral Data. (PDF) [file pone.0098977.s001.pdf]

Table S1: Behavioral Data

ID	Assay_No	Smoker	Age	Testosterone-level	Mediansplit
1	1	no	28	119,908	0
2	1	no	23	210,57	1
3	1	no	19	139,413	1
4	1	no	21	117,218	0
5	1	yes	21	153,544	1
6	1	no	23	207,254	1
7	1	no	25	115,209	0
8	1	yes	24	101,134	0
9	1	no	21	107,417	0
10	1	no	28	108,268	0
11	1	no	22	130,893	1
12	1	no	18	106,015	0
13	1	no	25	147,186	1
14	1	no	29	67,703	0
15	1	no	24	104,359	0
16	1	no	30	57,074	0
17	1	yes	26	163,296	1
18	1	no	26	120,874	0
19	1	no	21	90,902	0
20	1	no	27	76,518	0
21	1	yes	30	159,118	1
22	1	no	25	95,499	0
23	1	no	20	170,839	1
24	1	no	20	138,837	1
25	1	no	26	130,36	1
26	1	no	20	132,508	1
27	1	yes	24	130,537	1
28	1	no	25	80,692	0
29	1	yes	30	165,92	1
30	1	no	24	123	1
31	2	no	29	91,203	0
32	2	no	25	72,321	0
33	2	no	18	225,019	1
34	2	no	25	61,872	0
35	2	no	28	117,575	1
36	2	no	26	112,726	1
37	2	no	19	157,535	1
38	2	yes	23	100,21	0
39	2	no	24	77,992	0
40	2	no	21	122,01	1
41	2	yes	32	112,726	1
42	2	no	26	109,744	0
43	2	no	20	130,402	1
44	2	no	29	174,236	1
45	2	yes	29	177,544	1
46	2	yes	27	82,573	0
47	2	yes	27	114,029	1
48	2	no	24	87,254	0
49	2	yes	26	101,765	0
50	2	no	25	97,165	0

ID	NeutralContext_ingroup_Unfair (%_rejection)	NeutralContext_ingroup_Fair (%_rejection)	NeutralContext_antagonistic_outgroup_Unfair (%_rejection)	NeutralContext_antagonistic_outgroup_Fair (%_rejection)
1	60	0	80	0
2	100	0	100	75
3	60	0	100	0
4	80	0	100	50
5	40	0	100	0
6	0	0	100	0
7	40	0	80	0
8	100	0	100	25
9	100	25	100	25
10	60	0	100	50
11	40	0	100	0
12	100	25	100	25
13	60	25	100	100
14	100	0	100	25
15	0	0	100	100
16	100	25	100	0
17	20	0	60	0
18	80	0	80	0
19	0	25	100	100
20	80	0	80	0
21	100	0	80	0
22	0	0	100	75
23	100	0	100	0
24	40	0	40	0
25	100	0	100	0
26	40	0	100	25
27	100	0	100	25
28	100	0	80	0
29	0	0	0	0
30	100	0	100	0
31	0	0	0	0
32	20	0	100	25
33	80	0	80	0
34	0	0	20	0
35	100	0	100	25
36	40	0	100	100
37	20	0	40	0
38	100	0	100	100
39	0	0	0	0
40	0	0	100	25
41	100	0	100	25
42	80	0	100	0
43	0	0	0	0
44	80	0	40	0
45	0	0	100	100
46	80	0	100	25
47	0	0	0	0
48	80	0	100	25
49	0	0	40	0
50	80	0	100	25

ID	NeutralContext_neutral_outgroup_Unfair (%_rejection)	NeutralContext_neutral_outgroup_Fair (%_rejection)	NeutralContext_unknown_outgroup_Unfair (%_rejection)	NeutralContext_unknown_outgroup_Fair (%_rejection)
1	80	0	80	0
2	100	0	100	25
3	60	0	80	0
4	80	0	100	0
5	80	0	100	0
6	100	0	100	0
7	60	0	60	0
8	100	25	100	25
9	100	25	100	25
10	100	0	100	0
11	60	0	60	0
12	100	25	100	33,333332
13	100	25	100	0
14	100	0	100	0
15	0	0	80	100
16	60	25	100	0
17	40	0	40	0
18	80	0	80	0
19	100	25	100	25
20	80	0	80	0
21	80	0	80	0
22	60	0	20	0
23	100	0	100	0
24	40	0	40	0
25	100	0	80	0
26	40	0	80	25
27	80	25	100	25
28	75	0	80	0
29	0	0	0	0
30	100	25	100	25
31	0	0	0	0
32	80	0	60	0
33	60	0	80	0
34	20	0	0	0
35	100	25	100	0
36	60	0	60	0
37	40	0	40	0
38	100	0	100	100
39	0	0	0	0
40	40	0	60	0
41	100	25	100	0
42	80	0	100	0
43	0	0	0	0
44	60	0	60	0
45	0	0	40	0
46	100	0	100	0
47	0	0	0	0
48	100	0	100	25
49	40	0	40	0
50	100	0	100	0

ID	CompetitiveContext_ingroup_Unfair (%_rejection)	CompetitiveContext_ingroup_Fair (%_rejection)	CompetitiveContext_antagonistic_outgroup_Unfair (%_rejection)	CompetitiveContext_antagonistic_outgroup_Fair (%_rejection)
1	0	0	80	0
2	0	0	80	100
3	0	0	100	50
4	0	0	100	100
5	0	0	100	0
6	0	0	100	25
7	20	0	60	25
8	0	0	80	25
9	100	25	100	25
10	0	0	100	100
11	0	0	100	25
12	0	0	100	25
13	0	0	100	100
14	0	0	100	25
15	0	0	100	100
16	100	0	80	25
17	0	0	80	0
18	0	0	100	25
19	20	0	100	0
20	20	0	80	0
21	0	0	100	50
22	100	25	100	25
23	20	0	100	25
24	0	0	100	50
25	0	0	100	100
26	0	0	0	0
27	20	0	100	25
28	0	0	100	25
29	0	0	100	25
30	0	0	100	100
31	0	0	100	100
32	0	0	80	25
33	0	0	100	0
34	0	0	100	0
35	0	0	100	25
36	20	0	100	100
37	0	0	100	0
38	0	0	100	25
39	0	0	80	0
40	0	0	100	25
41	80	0	100	50
42	0	0	100	0
43	0	0	100	25
44	0	0	20	0
45	0	0	100	75
46	0	0	100	0
47	0	0	100	100
48	80	0	100	25
49	0	0	100	25
50	0	0	100	0

ID	CompetitiveContext_neutral_outgroup_Unfair (%_rejection)	CompetitiveContext_neutral_outgroup_Fair (%_rejection)	CompetitiveContext_unknown_outgroup_Unfair (%_rejection)	CompetitiveContext_unknown_outgroup_Fair (%_rejection)
1	80	0	100	0
2	100	100	100	100
3	100	75	100	50
4	100	25	100	0
5	80	0	100	0
6	100	25	100	25
7	60	0	60	0
8	80	0	100	0
9	100	25	100	25
10	100	100	80	75
11	100	25	100	25
12	100	25	100	25
13	100	100	100	100
14	100	0	100	0
15	100	100	100	100
16	100	0	100	0
17	40	0	80	0
18	100	25	100	25
19	100	0	100	25
20	100	0	100	0
21	0	0	100	0
22	100	25	100	25
23	100	25	100	25
24	20	0	100	50
25	100	75	100	75
26	0	0	0	0
27	100	25	100	25
28	100	25	100	25
29	100	25	100	25
30	100	100	100	100
31	100	100	100	75
32	80	0	100	0
33	100	0	100	0
34	80	25	100	0
35	100	25	100	25
36	100	0	100	0
37	100	25	100	0
38	100	25	100	25
39	80	0	100	0
40	100	0	100	25
41	100	50	100	50
42	100	0	100	0
43	100	25	100	25
44	0	0	0	0
45	80	0	100	0
46	100	0	80	0
47	100	100	100	100
48	100	25	100	25
49	100	25	100	0
50	100	0	60	0

ID	Points_NeutralContext_ingroup	Points_NeutralContext_antagonistic_outgroup	Points_NeutralContext_neutral_outgroup	Points_NeutralContext_unknown_outgroup
1	24	22	22	22
2	19	5	19	15
3	24	19	24	22
4	22	10	22	19
5	26	19	22	19
6	28	19	19	19
7	26	21	24	24
8	19	15	15	15
9	15	15	15	15
10	22	10	19	19
11	26	19	24	24
12	15	15	15	10
13	20	0	15	19
14	19	15	19	19
15	26	0	28	3
16	15	19	20	19
17	27	24	26	26
18	22	22	22	22
19	23	0	14	14
20	22	22	22	22
21	19	22	21	22
22	28	5	24	26
23	19	19	19	19
24	26	26	26	26
25	19	19	19	22
26	24	15	25	16
27	19	15	17	15
28	19	22	22	22
29	28	28	28	28
30	19	19	15	14
31	28	28	28	28
32	22	19	22	19
33	22	26	24	24
34	28	0	28	23
35	22	15	19	19
36	28	28	28	28
37	22	15	19	15
38	28	25	25	25
39	22	15	19	19
40	27	15	20	23
41	22	22	24	22
42	28	25	26	28
43	19	15	15	19
44	26	0	24	24
45	27	26	26	26
46	19	0	19	0
47	28	28	28	28
48	28	15	23	24
49	19	15	15	19
50	28	28	28	28

ID	Points_CompetitiveContext_ingroup	Points_CompetitiveContext_antagonistic_outgroup	Points_CompetitiveContext_neutral_outgroup	Points_CompetitiveContext_unknown_outgroup
1	28	22	22	19
2	28	1	0	0
3	28	10	5	10
4	28	0	15	19
5	28	19	22	19
6	28	15	15	15
7	26	19	24	24
8	28	17	20	19
9	15	15	15	15
10	28	0	0	6
11	28	15	15	15
12	28	15	15	15
13	28	0	0	0
14	28	15	19	19
15	28	0	0	0
16	19	15	19	19
17	28	22	25	22
18	28	15	15	15
19	27	19	19	15
20	27	22	19	19
21	28	10	28	19
22	15	15	15	15
23	25	15	15	15
24	28	10	26	10
25	28	0	5	5
26	27	28	28	28
27	25	15	15	15
28	28	15	15	15
29	28	15	15	15
30	28	0	0	0
31	28	0	0	5
32	28	19	19	19
33	28	27	28	28
34	28	5	21	19
35	28	19	19	22
36	28	0	0	0
37	20	15	15	15
38	28	15	15	19
39	28	19	19	23
40	28	18	21	19
41	28	19	19	19
42	28	19	18	19
43	28	15	15	15
44	27	0	19	19
45	28	19	15	19
46	28	15	15	15
47	28	22	21	19
48	28	14	19	15
49	22	10	9	10
50	28	15	15	15

ID	EmailOffer_To_ingroup	EmailOffer_To_antagonistic_outgroup	EmailOffer_To_neutral_outgroup	EmailOffer_To_unknown_outgroup
1				
2	5	3	5	5
3				
4	5	2	3	3
5	5	1	3	1
6				
7				
8				
9	4	4	4	4
10	1	1	3	5
11	5	0	3	0
12				
13	5	3	5	4
14				
15	5	2	4	2
16				
17				
18	4	4	4	4
19	5	0	3	0
20	4	1	2	3
21	5	1	4	1
22	5	0	3	2
23	5	4	4	4
24	5	1	4	5
25	5	0	3	4
26	5	1	2	1
27	5	5	5	5
28	4	4	4	4
29	5	5	5	5
30	4	1	3	4
31	5	4	3	5
32	5	2	3	4
33	5	3	3	3
34	5	3	4	4
35	5	5	5	5
36	5	5	5	5
37	3	3	3	3
38	5	1	3	1
39	5	3	3	3
40	5	1	3	2
41	5	4	4	4
42	5	4	5	5
43	5	1	3	2
44	6	3	2	0
45	10	0	3	0
46	5	3	4	4
47	5	2	2	2
48	5	3	4	4
49	5	2	3	1
50	5	2	3	4
